# Molecular Defects of the Disease-Causing Human Arrestin-1 C147F Mutant

**DOI:** 10.1167/iovs.17-22180

**Published:** 2018-01

**Authors:** Sergey A. Vishnivetskiy, Lori S. Sullivan, Sara J. Bowne, Stephen P. Daiger, Eugenia V. Gurevich, Vsevolod V. Gurevich

**Affiliations:** 1 Department of Pharmacology, Vanderbilt University, Nashville, Tennessee, United States; 2 Human Genetics Center, School of Public Health, The University of Texas Health Science Center, Houston, Texas, United States

**Keywords:** arrestin, retinitis pigmentosa, photoreceptors, protein stability

## Abstract

**Purpose:**

The purpose of this study was to identify the molecular defect in the disease-causing human arrestin-1 C147F mutant.

**Methods:**

The binding of wild-type (WT) human arrestin-1 and several mutants with substitutions in position 147 (including C147F, which causes dominant retinitis pigmentosa in humans) to phosphorylated and unphosphorylated light-activated rhodopsin was determined. Thermal stability of WT and mutant human arrestin-1, as well as unfolded protein response in 661W cells, were also evaluated.

**Results:**

WT human arrestin-1 was selective for phosphorylated light-activated rhodopsin. Substitutions of Cys-147 with smaller side chain residues, Ala or Val, did not substantially affect binding selectivity, whereas residues with bulky side chains in the position 147 (Ile, Leu, and disease-causing Phe) greatly increased the binding to unphosphorylated rhodopsin. Functional survival of mutant proteins with bulky substitutions at physiological and elevated temperature was also compromised. C147F mutant induced unfolded protein response in cultured cells.

**Conclusions:**

Bulky Phe substitution of Cys-147 in human arrestin-1 likely causes rod degeneration due to reduced stability of the protein, which induces unfolded protein response in expressing cells.

Visual arrestin-1 was first discovered for its ability to bind light-activated phosphorylated rhodopsin (P-Rh*)^1^ and quench rhodopsin signaling,^2^ apparently due to direct competition of active rhodopsin with visual G protein transducin.^3,4^ The function of arrestin in the termination of rhodopsin signaling is critical: the lack of arrestin,^5^ the absence of rhodopsin kinase,^6,7^ the absence^8,9^ or insufficient number^10^ of phosphorylation sites in rhodopsin preclude high-affinity arrestin-1 binding^11,12^ as well as cause visual defects in mice and humans. However, a previously identified loss-of-function mutation in arrestin-1^13^ is recessive, as the other perfectly normal allele is sufficient for function, in line with our earlier finding that as little as 4% of wild-type (WT) arrestin expression level is sufficient for maintaining rod health in mice.^14^ It is interesting that recently identified C147F mutation in the human arrestin-1 is dominant and causes retinitis pigmentosa.^15^ Thus, loss of function cannot explain the mechanism of action of this mutant arrestin-1. Cys147 in the human arrestin-1 is homologous to the Cys143 of the bovine protein, which in crystal structures^16,17^ is placed in the closely packed area in the central crest of the two-domain arrestin-1 molecule between receptor-binding “finger” loop and “139-loop.”^18–22^ Thus, this mutation can potentially affect at least two aspects of arrestin-1 function, rhodopsin binding and folding. Here we set out to determine the molecular defects of the C147F mutant that might underlie human disease.

## Methods

### Materials

[γ-^32^P]ATP, [^14^C]leucine, and [^3^H]leucine were from Perkin-Elmer (Waltham, MA, USA). All restriction and DNA-modifying enzymes were from New England Biolabs (Ipswich, MA, USA). Rabbit reticulocyte lysate was from Ambion (Austin, TX, USA), and SP6 RNA polymerase was prepared as described.^23^

### Mutagenesis and Plasmid Construction

For in vitro transcription, the human arrestin-1 cDNA was subcloned into pGEM2 (Promega, Madison, WI, USA) with “idealized” 5-UTR^23^ between *Nco*I and *Hind*III sites, as described for the bovine^11^ and mouse arrestin-1.^24^ All mutations were introduced in the transcription construct by PCR and verified by dideoxy sequencing. For *Escherichia coli* expression, cDNA encoding WT human arrestin-1 and its C147F mutant was subcloned into pTrcHisB vector between *Nco*I and *Hind*III sites (this eliminates His6-tag so that unmodified protein is expressed), as described.^25^ Test expression in 3 mL bacterial culture, cell lysis, and separation of soluble protein from cell debris were performed, as described.^26^ In vitro transcription, translation, preparation of phosphorylated and unphosphorylated rhodopsin were performed as described recently.^27,28^

### Electrophoresis, Autoradiography, and Western Blotting

Cell-free translated protein labeled with ^14^C-Leu and ^3^H-Leu was subjected to SDS-PAGE in 10% polyacrylamide (PAAG) gel, which was then stained with a reagent (GelCode Blue Coomassie-based; Thermo Scientific, City State, USA), destained with water, and soaked in 10% 2,5-diphenyloxazole (PPO) in acetic acid. PPO was then precipitated within the gel by immersing it in water, and radioactive bands were visualized by autoradiography with X-ray film. Aliquots of the lysates of arrestin-expressing *E. coli* and supernatants after high-speed centrifugation of these lysates containing soluble proteins were subjected to SDS-PAGE in 10% PAAG gel and transferred to polyvinylidene fluoride (PVDF) membranes. Arrestin bands were visualized using pan-arrestin rabbit F431 primary and horseradish peroxidase (HRP)-conjugated anti-rabbit secondary antibodies, followed by incubation with reagent (WestPico ECL; Thermo Scientific) and exposure to X-ray film.

Direct binding assay was performed, as described.^27^ Briefly, 1 nM arrestin-1 (50 fmol) was incubated with 0.3 μg P-Rh* or Rh* (11 pmol, yielding final concentration of 0.22 μM) in 50 μL of 50 mM Tris-HCL, pH 7.4; 100 mM potassium acetate; 1 mM EDTA; 1 mM dithiothreitol for 5 minutes at 37°C (or other indicated temperature) under room light. Samples were cooled on ice, whereupon bound and free arrestin-1 were separated at 4°C by gel filtration on a 2-mL column of Sepharose 2B-CL. Arrestin-1 eluting with rhodopsin-containing membranes was quantified by liquid scintillation counting. Nonspecific binding, determined in samples where rhodopsin was omitted, was subtracted.

### In Vitro Arrestin Stability Assay

Translated radiolabeled arrestin-1 was incubated for 1 or 2 hours at 42°C, or for up to 24 hours at 37°C, and cooled on ice. The binding of arrestin-1 to P-Rh* in these samples was compared to that of control sample kept on ice, as described above; 1 nM arrestin-1 (50 fmol per sample) was used, as in the standard direct binding assay.

### Cell Culture

Photoreceptor-derived 661W 29 cells,^29^ which are more appropriate for studies of visual arrestin-1 than most cultured cell lines, were used to express WT human arrestin-1 and its C147F mutant. These cells were originally isolated from a transgenic mouse line expressing SV40 T-antigen under controls of human interphotoreceptor retinol-binding protein promoter.^29^ The cells were cultured in Dulbecco's modified Eagle's medium containing 10% fetal bovine serum. The cells were transfected with constructs containing the coding sequence of the human arrestin-1 or its C147F mutant in pcDNA3 using transfection reagent (Trans-Hi; FormuMax Scientific, Sunnyvale, CA, USA). Empty pcDNA3 vector was used as control. Untransfected 661W cells treated for 1 hour with 100 μM tunicamycin, which inhibits *N*-linked glycosylation, or 1 μM thapsigargin (30 minutes), which inhibits endoplasmic reticulum (ER) Ca^2+^ ATPase, were used as positive controls for ER stress and unfolded protein response (UPR). The cells were transfected 48 hours prior to harvesting or treated with thapsigargin or tunicamycin right before harvesting. The cells were lysed using 150 μL of lysis buffer (Ambion) per well of a 12-well plate. After brief sonication (10 seconds, 10% maximum power) using a sonic dismembrator (model 500; Fisher Scientific, Pittsburgh, PA, USA), the protein in the lysates was measured using Bradford method (Bio-Rad, Hercules, CA, USA). The protein was precipitated by the addition of nine volumes of methanol and pelleted by centrifugation for 10 minutes at 13,000 rpm (Eppendorf MiniSpin centrifuge). The pellet was washed with 1 mL 90% methanol, dried, and dissolved in SDS sample buffer (Sigma-Aldrich Corp., St. Louis, MO, USA) at 0.5 μg/μL.

### Western Blotting

The protein was subjected to SDS-PAGE in 10% (GAPDH and arrestin) or 8% (GRP78/BiP) acrylamide gel and transferred to PVDF membrane. The proteins were visualized using the following primary antibodies: anti-GAPDH (loading control, mouse, 1:1000), anti-arrestin (F431 rabbit polyclonal,^26^ 1:5000), and anti-BIP (rabbit polyclonal, 1:1000 dilution; Cell Signaling, Danvers, MA, USA). Blots were incubated with primary antibodies overnight at 4°C, washed three times with TBS-0.1% Tween-20, then incubated with corresponding HRP-conjugated secondary antibodies (1:5000; Jackson ImmunoResearch Laboratories, Inc., West Grove, PA, USA). The bands were developed using ECL pico reagent (Thermo Fisher Scientific) (5 minutes) and visualized by exposure to X-ray film (VWR, Radnor, PA, USA). The signal was quantified using VersaDoc with QuantityOne software (Bio-Rad).

### Statistical Analysis

Statistical analysis was performed by ANOVA using analysis software (StatView SAS Institute, Cary, NC, USA).

## Results and Discussion

**Figure 1:**
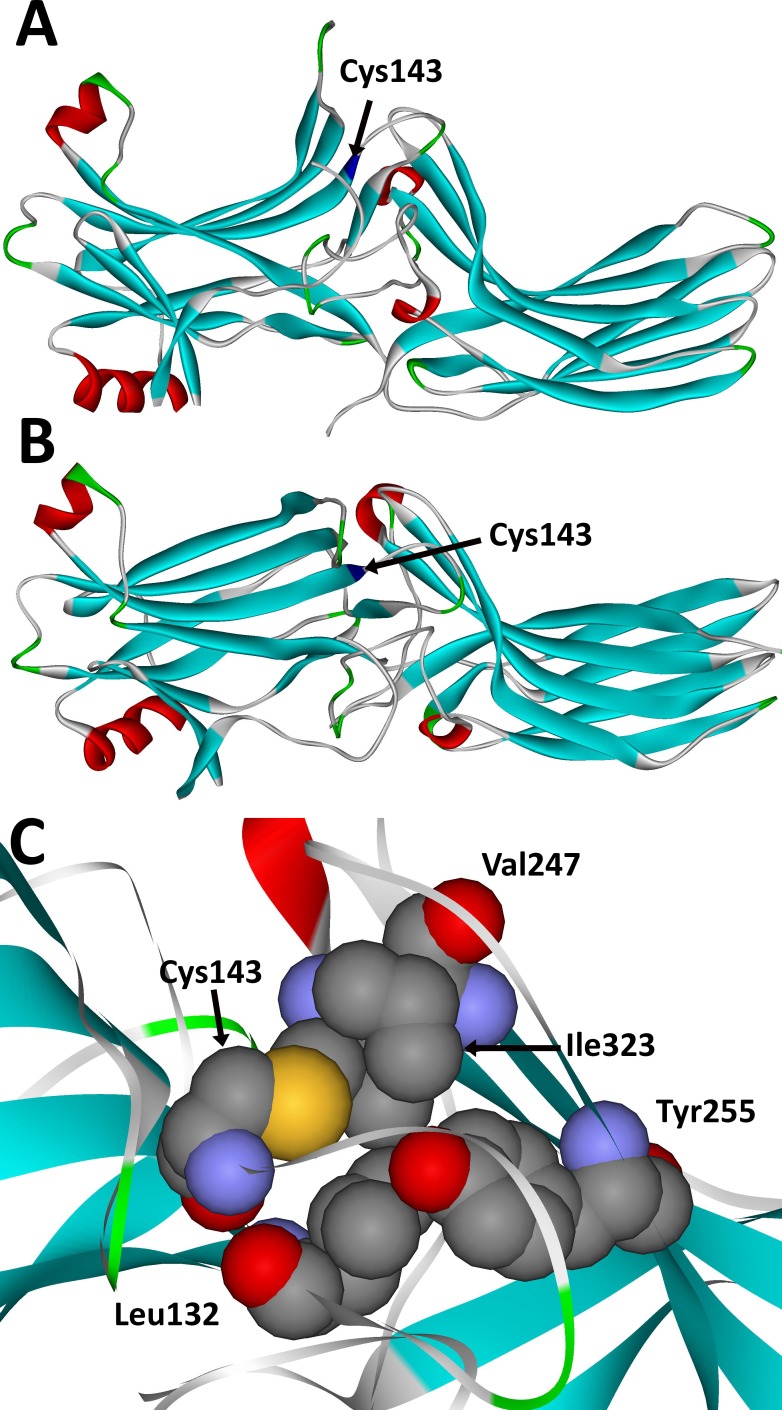
The localization of cysteine-147 in the arrestin-1 protein. (A, B) Crystal structure of the bovine arrestin-1 (PDB 1CF1^17^) colored by secondary structure: β-strands, blue; β-turns, green; α-helices, red. Cys-147 (Cys-143 in bovine arrestin-1) is blue and indicated. A view from the side (A) and receptor-binding side (B) of the molecule is shown. Note that Cys-147 is located between the two loops in the central crest of the receptor-binding side of the molecule. Both the finger loop and 139-loop were implicated in receptor binding by site-directed spin labeling/EPR,^18,20^ mutagenesis,^22^ and crystallography.^19,21,36^ (C) Blow-up of the view from the receptor-binding side showing bovine Cys-143 and its immediate neighbors, Leu-132, Val-247, Tyr-255, and Ile-323 in the bovine arrestin-1, all of which are conserved in the human protein. Note the close packing of Cys-143, suggesting that a much larger side chain of phenylalanine in this position would not fit and therefore would likely cause a shift of neighboring residues and elements containing them.

Arrestin-1,^16,17^ as well as all other vertebrate arrestin isoforms,^30–33^ is an elongated two-domain molecule with relatively few contacts between domains (Figs. 1A, 1B), perfectly suited for proposed global conformational change upon receptor binding,^34,35^ which was recently confirmed by the structure of the arrestin-1–rhodopsin complex.^19,36^ Cys-143 in bovine arrestin-1, homologous to Cys-147 in human protein, is not exposed; it is located between the two loops in the central crest of the receptor-binding arrestin surface (Figs. 1A, 1B), where in the bovine protein it is closely packed with several hydrophobic partners: Leu132, Val247, Tyr255, and Ile323 (Fig. 1C).^17^ Thus, the substitution of a relatively small Cys side chain with a bulky Phe could potentially lead to misfolding and/or temperature-sensitive unfolding of the protein. There are two differences between Cys and Phe: the presence of a functional SH group in the former, but not in the latter, and the size of the side chain. As the vertebrate visual arrestin-1 was not reported to undergo posttranslational modifications,^37^ where the SH group could have played a role, we focused on the size of the side chain and generated mutants containing small Ala, medium-sized Val, and bulky Leu, Ile, and Phe residues in position 147. In case of the bovine arrestin, we previously found that Ala or Val in position 143 do not significantly change functional characteristics of WT arrestin-1.^18,38–40^

**Figure 2:**
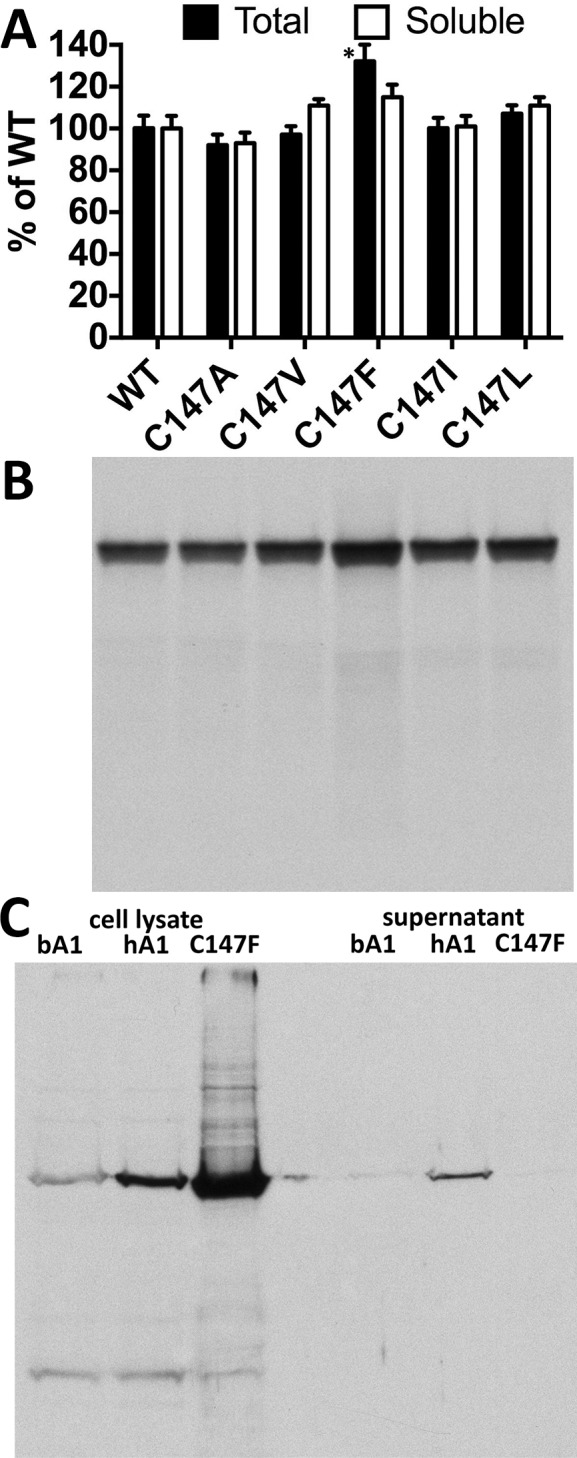
Near-normal expression and initial folding of the arrestin-1-C147F mutant in cell-free translation, but not in E. coli. (A) The expression levels in cell-free translation (black bars) and the amounts of indicated forms of the human arrestin-1 that remain soluble after high-speed centrifugation (white bars) are shown as percentage of corresponding values of WT human arrestin-1. (The means ± SD of two experiments, each performed in duplicate, are shown. The expression of WT human arrestin-1 was 15.9 ± 0.9 fmol/μL; after high-speed centrifugation, 13.6 ± 0.8 fmol/μL remained in the supernatant). The absolute values (before normalization) were analyzed by 1-way ANOVA with protein as a factor, using Bonferroni post hoc test with correction for multiple comparisons. *P < 0.05, as compared to WT human arrestin-1. The fraction of the soluble (properly folded) arrestin-1-C147F was lower than that of all other forms. (B) Radiolabeled in vitro translated proteins (5 μL) were mixed with 15 μL of SDS sample buffer and subjected to SDS-PAGE in 10% acrylamide gel. The gel was stained with Coomassie blue (Pierce, Pittsburgh, PA, USA), destained by several washes with water, and soaked in acetic acid with 20% (wt/vol) PPO (2,5-diphenyloxazole). PPO was precipitated by immersion of the gel into water. The gel was dried and exposed to X-ray film for 3 days, revealing translated protein bands by autoradiography. (C) The WT bovine (bA1), WT human (hA1), and human C147F mutant (C147F) were expressed in E. coli. Equal fractions of cell lysates and supernatants after high-speed centrifugation were subjected to SDS-PAGE, transferred to PVDF membrane, and visualized using rabbit pan-arrestin F431 primary, HRP-conjugated anti-rabbit secondary antibodies, and SuperSignal ECL reagent.

Proper folding is necessary for protein functionality. Protein yield in cell-free translation and the fraction of translated protein that remains soluble after high-speed centrifugation are good indicators of proper folding.^41^ Cell-free translation is a relatively demanding system, as the cytoplasm of rabbit reticulocytes is diluted several-fold^42^ so that the concentrations of all chaperones that assist protein folding are significantly reduced. Thus, first we compared the yields of WT and mutant forms of the human arrestin-1 in cell-free translation as well as the fraction of soluble arrestin after centrifugation at ∼600,000*g* for 1 hour (Figs. 2A, 2B). We did not detect greater than 33% difference in the expression levels of WT and mutant forms of the human arrestin-1. The fraction of these proteins that remained soluble after centrifugation was also similar, although the fraction of the soluble C147F mutant (∼75%) was lower than that of the others (85% or greater) (Fig. 2A). Still, the level of translated radiolabeled C147F mutant after centrifugation was similar to that of WT protein (Fig. 2B). These data excluded gross misfolding as the likely reason for pathology caused by the C147F mutation in human patients.^15^ In all cases, the band with the expected molecular weight of human arrestin-1 contained >95% of radiolabeled protein (Fig. 2B), indicating that WT and mutant forms of human arrestin-1 did not undergo proteolysis.

WT visual arrestin-1 from different species (bovine, mouse, and human) successfully folds even in *E. coli*, which has fewer chaperones than eukaryotic cells and can be purified from expressing bacteria in large quantities.^25,26^ Therefore, next we compared the expression of human arrestin-1 C147F mutant with the WT human and bovine proteins in *E. coli*, as well as the soluble fraction of these proteins (Fig. 2C). To this end, we performed test expression in 3 mL of bacterial culture. After 4-hour induction with isopropyl β-d-1-thiogalactopyranoside at 30°C, the cells were pelleted and lysed following standard procedure,^26^ then cell debris along with inclusion bodies (where bacteria place misfolded or denatured proteins) and aggregates were separated from soluble proteins by centrifugation at ∼600,000*g* for 1 hour. Western blotting of the aliquots of cell lysates and supernatants with rabbit pan-arrestin F431 antibody^26^ revealed that C147F mutant expresses at a higher level than does WT human arrestin-1, but practically all of it is insoluble in contrast to WT bovine and human arrestin-1 (Fig. 2C). Thus, C147F mutant either does not fold as easily as WT protein in the absence of mammalian chaperones or readily denatures and aggregates. Only a small fraction of immunoreactive material was present as smaller molecular weight species, which were visible in all cases (Fig. 2C), suggesting that in vivo proteolysis in *E. coli* is not a major issue. The bands with larger molecular weight, particularly the band at the top of the resolving gel (Fig. 2C), are more prominent in case of C147F mutant, suggesting that it aggregates more than WT human arrestin-1.

**Figure 3:**
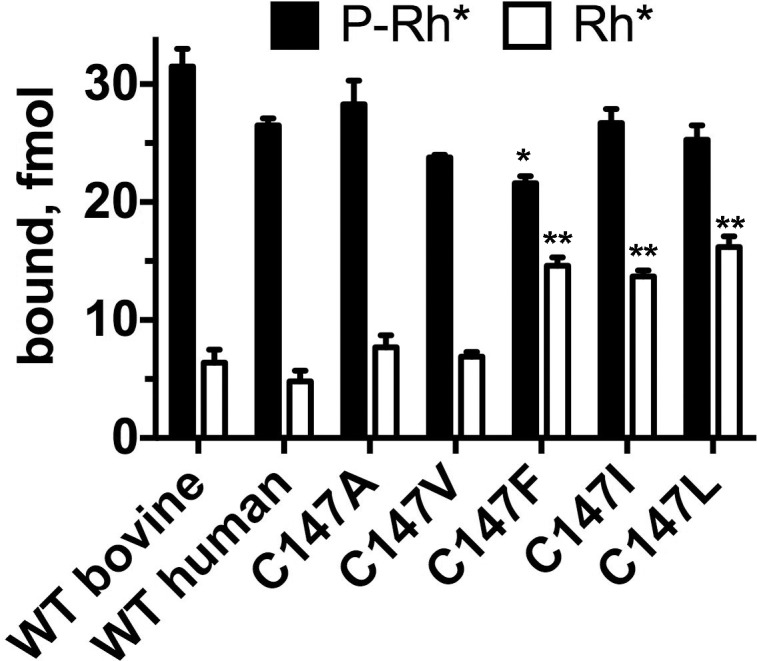
Reduced selectivity of the arrestin-1-C147F and other mutants with bulky hydrophobic residues in 147 position. The binding of the WT bovine and human arrestin-1 and indicated mutants is shown. The bars are colored, as follows: black, P-Rh*; white, Rh*. The means ± SD of two experiments each performed in duplicate are shown. The P-Rh* and Rh* binding was analyzed separately by 1-way ANOVA with protein as a factor, using Bonferroni post hoc test with correction for multiple comparisons. *P < 0.05; **P < 0.01, as compared to WT human arrestin-1.

As the key function of arrestin is specific binding to the active phosphorylated rhodopsin,^1,12^ we expressed WT and mutant human arrestin-1 with several substitutions in position 147 in cell-free translation in the presence of radioactive amino acids and determined the binding of synthesized radiolabeled proteins to purified P-Rh* in our standard in vitro binding assay.^12^ Since rhodopsin used in this assay was purified from cow eyes, we used bovine arrestin-1 as a positive control (Fig. 3). We confirmed that the bovine rhodopsin binds the human arrestin-1 essentially as well as the bovine arrestin-1. We previously found this to be the case for the mouse arrestin-1,^24,25^ which reflects high conservation of both rhodopsin^43^ and arrestin-1^44,45^ in mammals. Although the human arrestin-1 C147F mutant demonstrated the lowest P-Rh* binding in the group (Fig. 3), it differed from WT by less than 20%. As mouse retinas expressing only 12% and even 4% of WT level of arrestin-1 were perfectly healthy,^14^ this modest reduction in binding is unlikely to cause photoreceptor health problems in vivo. Moreover, in vitro direct binding assay was performed at 1 nM arrestin-1, whereas the concentration of arrestin-1 in rods is six orders of magnitude higher, exceeding 2 mM.^14,46^ Another critical functional characteristic of arrestin-1 is high selectivity for P-Rh*: The binding to P-Rh* exceeds the binding to unphosphorylated Rh* >5-fold.^1,12,47^ Therefore, next we tested Rh* binding of all forms of arrestin-1. While the binding of C147A and C147V mutants to Rh* was only marginally increased compared to WT (in agreement with our earlier results with the bovine protein),^18,38–40^ substitution of Cys-147 with bulkier Leu, Ile, and Phe increased Rh* binding more than 2-fold (Fig. 3). Two lines of evidence suggest that this loss of selectivity for the phosphorylated receptor is unlikely to cause severe retinal degeneration. First, several mammalian species with healthy retinas express the phosphorylation-independent arrestin-1 splice variant p44 that demonstrates significantly higher binding to Rh* than does full-length protein.^48,49^ Second, we previously did not detect retinal degeneration or visual impairment in mice transgenically expressing engineered phosphorylation-independent arrestin-1 mutants,^14,50^ at least at low to physiological levels.^51,52^ The dominant nature of the C147F mutant in humans is also incompatible with the damage being done by its reduced selectivity because the second allele in patients encodes WT arrestin-1. In mice coexpression of WT arresin-1 partially protects against the damage by the enhanced mutant expressed even at high levels and fully protects against lower levels of the phosphorylation-independent mutant.^51^

**Figure 4:**
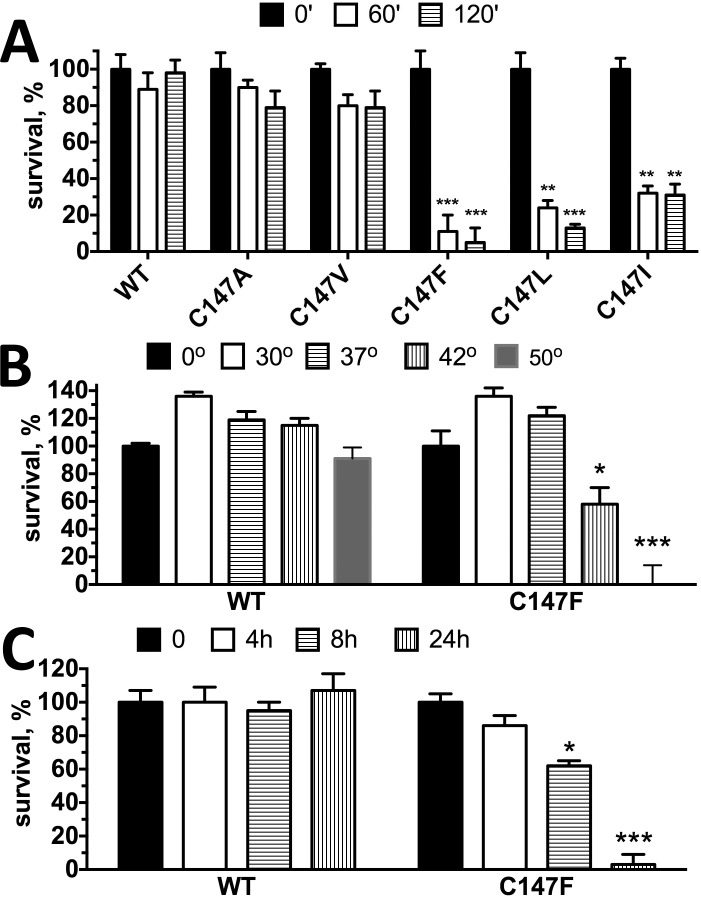
Phenylalanine and other bulky hydrophobic residues in position 147 reduce the preservation of functionality of the human arrestin-1 at elevated temperature. (A) The binding of the WT human arrestin-1 and indicated mutants to P-Rh* after incubation for 1 (60 minutes) and 2 hours (120 minutes) at 42°C (control proteins were kept on ice). The means ± SD of two independent experiments performed in duplicate are shown. The data for each time point were analyzed separately by 1-way ANOVA with protein as a factor, using Bonferroni post hoc test with correction for multiple comparisons. **P < 0.01; ***P < 0.001, as compared to WT human arrestin-1. (B) The WT human arrestin-1 and C147 mutant were incubated for 30 minutes at indicated temperatures, whereupon the binding of these arrestins to P-Rh* was measured in a standard assay. *P < 0.05; ***P < 0.001, as compared to WT human arrestin-1. (C) The WT human arrestin-1 and C147 mutant were incubated for indicated times at 37°C, and the binding of these arrestins to P-Rh* was measured in a standard assay. *P < 0.05; ***P < 0.001, as compared to WT human arrestin-1.

However, our structure-function studies suggest that increased Rh* binding is often associated with reduced thermal stability of the protein.^20,22^ Therefore, next we tested the functional integrity of WT and mutant human arrestin-1 forms at elevated temperature. To make this test rigorous, we incubated WT human arrestin-1 and mutants at 42°C for 1 and 2 hours, and then tested specific binding to P-Rh* (Fig. 4A). We found that the WT human arrestin-1 is as stable as its bovine^50^ and mouse^24^ homologues: There was virtually no loss of activity even after 2 hours at 42°C. The replacement of Cys-147 with alanine or valine only marginally reduced preservation of function of the protein, in agreement with our previous experience with bovine arrestin-1.^18,20^ However, C147F mutation had a very strong effect: this mutant largely lost the ability to specifically bind P-Rh* after just 1 hour (Fig. 4A), likely due to denaturing at higher temperature. Next, we compared functional survival of the WT human arrestin-1 and its C147F mutant after 30 minutes of incubation at temperatures from 0°C to 50°C, followed by standard P-Rh* binding for 5 minutes at 37°C (Fig. 4B). We found that while the WT arrestin-1 retains full functionality up to 42°C, and >80% even at 50°C, the C147F mutant loses almost half of its P-Rh* binding ability at 42°C and virtually all at 50°C (Fig. 4B). These data also suggest that thermal stability of the C147F mutant is severely compromised, as compared to the WT human arrestin-1. However, physiological human temperature is slightly lower than 37°C. Therefore, we compared functional survival of the WT human arrestin-1 and C147F mutant at this temperature (Fig. 4C) and found that WT retains full functionality for at least 24 hours, whereas the ability of the C147F mutant to bind P-Rh* is significantly reduced after 8 hours and completely gone after 24 hours (Fig. 4C). Thus, the C147F mutant denatures much faster than the WT protein at physiological temperature. Accelerated denaturing appears to be the most likely reason for the photoreceptor loss caused by this mutation in humans^15^ as arrestin-1 is a very abundant protein in both rods^14,46,53^ and cones,^54^ thus any acceleration of its denaturing would likely overwhelm the proteasome system and cause cell death via UPR.^55^ It is interesting that this loss of functional integrity is clearly related to the size of the residue in position 147, as substitutions of Cys-147 with relatively bulky Leu or Ile also cause progressive reduction of P-Rh* binding at 42°C, although the survival of these mutants at 1 hour was 2- to 3-fold higher than that of the C147F mutant (Fig. 4A).

**Figure 5:**
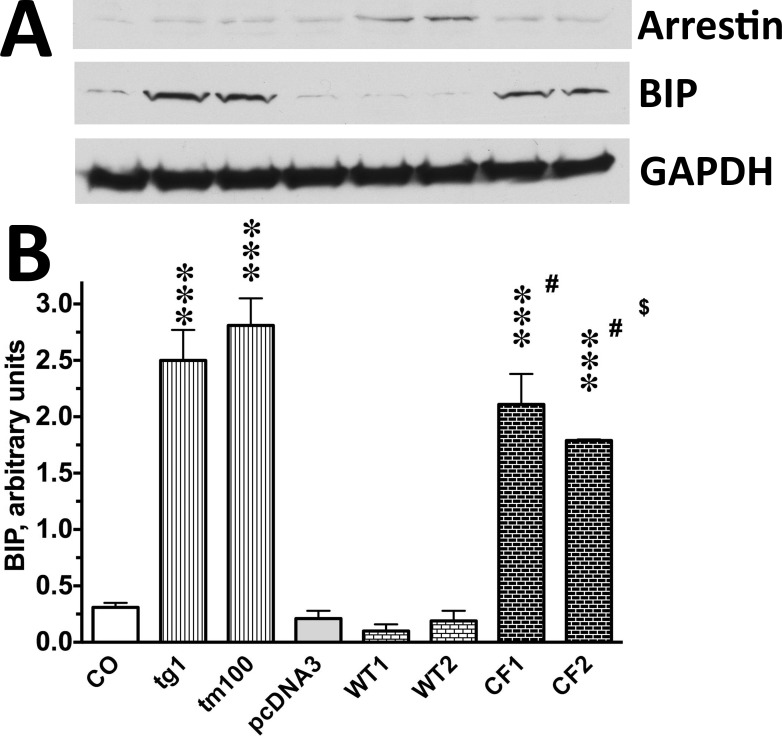
The C147F mutant, but not WT human arrestin-1, induces UPR in 661W cells. (A) Representative Western blots for indicated proteins are shown. For BIP and arrestin, 10 μg protein per lane was loaded; for GAPDH, the blots were stripped and reprobed with mouse anti-GAPDH antibody. (B) Quantification of BIP expression in control cells (CO) cells, cells treated with known inducers of UPR 1 μM of thapsigargin (tg1) or 100 μM tunicamycin (tm100), as well as cells transfected with empty vector (pcDNA3) or pcDNA3 encoding the WT human arestin-1 (DNA per well of six-well plate: 0.56 μg, WT1; 0.64 μg, WT2) or C147F mutant (DNA per well of six-well plate: 0.64 μg, CF1; 0.75 μg, CF2). The data are presented in arbitrary units. The intensity of the bands was normalized to the sum of intensities of all eight bands on the blot in each experiment (n = 3) and multiplied by 10. The data were analyzed by 1-way ANOVA (F(7,16) = 49.4, P < 0.0001), followed by Bonferroni's post hoc comparisons to corresponding controls (CO or pcDNA3). Statistical significance of the differences is shown, as follows: ***P < 0.001, #P < 0.001 to WT1/2, $P < 0.05 to tm100.

Accelerated loss of native folding is expected to induce UPR in expressing cells. Therefore, we tested whether this is the case using photoreceptor-derived cultured 661W cells^29^ expressing the WT human arrestin-1 or its C147F mutant (Fig. 5). As a positive control, we used 661W cells treated with 1 μM thapsigargin or 100 μM tunicamycin for 1 hour, the compounds that inhibit ER Ca^2+^ ATPase or *N*-linked glycosylation, respectively, thereby causing ER stress and UPR. As an indicator of UPR, we chose the increase in BiP/GRP78 expression,^56^ which is the first step in the UPR signaling cascade.^57^ We found that the expression of the WT human arrestin-1 does not increase BiP over the basal (control) level, whereas the expression of the C147F mutant significantly increases BiP expression. In cells expressing the C147F mutant, BiP was almost at the level observed in cells treated with tunicamycin or thapsigargin, even though achieved expression of the C147F mutant was much lower than that of WT protein (Fig. 5). The 661W cells are of photoreceptor origin and appear to express WT mouse arrestin-1 (Fig. 5) so that coexpression of the C147F mutant modeled the situation in human photoreceptors, where this mutant is coexpressed with the WT arrestin-1. Thus, arrestin-1-C147F clearly induces UPR (Fig. 5), indicating that it either does not fold properly or has much greater propensity to unfold than the WT human arrestin-1.

The three cysteines found in the *N*-domain of arrestin-1 of all species, as well as in other arrestin subtypes,^45^ were recently shown to play an important role in arrestin transition into high-affinity rhodopsin-binding conformation.^58^ In particular, Cys-147 in human arrestin-1, which corresponds to Cys-143 in bovine protein, is localized in a functionally and structurally critical position between the two loops in the central crest of the receptor-binding surface (Fig. 1) that are directly involved in rhodopsin binding and maintenance of the basal conformation. The finger loop (residues 68–78 in bovine protein, corresponding to residues 72–82 in human arrestin-1) was shown to directly engage the receptor by site-directed spin labeling^18,28^ and crystal structure of the arrestin-1–rhodopsin complex.^19,36^ In fact, the peptide representing the finger loop was cocrystallized with the activated rhodopsin, where it occupies the cavity between the cytoplasmic ends of the α-helices^59^ that appears upon rhodopsin activation.^60^ The Cys-143 in the bovine arrestin-1 (equivalent to Cys-147 in the human protein) is the first residue of the β-strand IX, right at its contact with the 139-loop, encompassing residues 121–142 in the bovine and corresponding residues 125–146 in the human protein.^17^ This loop was shown to move by up to 17 Å upon rhodopsin binding,^20^ and several residues in this loop were found to directly engage the receptor.^19,36^ It is interesting that shortening this loop greatly reduces the arrestin-1 selectivity for P-Rh* and the ability of the protein to maintain its basal conformation.^20,22^ Thus, destabilization of the basal conformation by the substitution of Cys-147 by a much bulkier phenylalanine, observed in vitro (Fig. 4) and in cultured cells (Fig. 5), is consistent with available structural data.

## Conclusions

Our characterization of the WT and mutant human arrestin-1 indicates that reduced protein stability underlies the pathogenesis and dominant nature of disease-causing C147F mutation in arrestin-1. Reduced stability was determined directly as accelerated loss of activity upon incubation at elevated temperature (Fig. 4), induction of UPR in cell culture (Fig. 5), as well as indirectly, as reflected in increased binding to a nonpreferred form of rhodopsin, light-activated unphosphorylated Rh* (Fig. 3). However, considering that many species express arestin-1 splice variant p44, which binds Rh* much better than full-length protein without any adverse consequences, this reduction in selectivity is unlikely to be the underlying cause of photoreceptor death. Collectively, our data suggest that the most likely cause of photoreceptor death in humans carrying C147F mutation in arrestin-1 is its accelerated loss of native folding, resulting in UPR, which causes cell stress and, ultimately, photoreceptor death in patients.
